# Gastric Cancer Image Classification: A Comparative Analysis and Feature Fusion Strategies

**DOI:** 10.3390/jimaging10080195

**Published:** 2024-08-10

**Authors:** Andrea Loddo, Marco Usai, Cecilia Di Ruberto

**Affiliations:** Department of Mathematics and Computer Science, University of Cagliari, Via Ospedale 72, 09124 Cagliari, Italy; m.usai84@studenti.unica.it (M.U.); cecilia.dir@unica.it (C.D.R.)

**Keywords:** computational pathology, histopathological imaging, gastric cancer, convolutional neural networks, machine learning, deep learning, feature extraction, feature combination

## Abstract

Gastric cancer is the fifth most common and fourth deadliest cancer worldwide, with a bleak 5-year survival rate of about 20%. Despite significant research into its pathobiology, prognostic predictability remains insufficient due to pathologists’ heavy workloads and the potential for diagnostic errors. Consequently, there is a pressing need for automated and precise histopathological diagnostic tools. This study leverages Machine Learning and Deep Learning techniques to classify histopathological images into healthy and cancerous categories. By utilizing both handcrafted and deep features and shallow learning classifiers on the GasHisSDB dataset, we conduct a comparative analysis to identify the most effective combinations of features and classifiers for differentiating normal from abnormal histopathological images without employing fine-tuning strategies. Our methodology achieves an accuracy of 95% with the SVM classifier, underscoring the effectiveness of feature fusion strategies. Additionally, cross-magnification experiments produced promising results with accuracies close to 80% and 90% when testing the models on unseen testing images with different resolutions.

## 1. Introduction

Gastric cancer is the fifth most prevalent cancer globally and the fourth leading cause of cancer-related deaths, with a global 5-year survival rate hovering around 20%. Despite significant research into the disease’s pathobiology, predicting its progression remains difficult, contributing to the persistently low survival rates. Furthermore, medical diagnostics’ intricate and time-consuming nature can lead to missing critical details during microscopic examinations, potentially resulting in misdiagnoses [1,2].

While recent advancements in computer technology, especially in Machine Learning (ML) and Deep Learning (DL), have enabled notable progress [3,4,5,6], there remain significant challenges [6,7]. Existing models often require extensive fine-tuning and customization to perform well in specific medical imaging tasks, which may not always be feasible in practical scenarios [3,6]. Additionally, the transferability and general applicability of features derived from pre-trained models to the medical domain are not well understood [6,8,9]. There is also a lack of comprehensive studies comparing the effectiveness of handcrafted (HC) features versus deep features across different classifiers in the context of gastric cancer histopathological images [4,6,9].

Creating computational tools that can automatically and accurately perform histopathological diagnoses is essential to addressing these challenges. In this study, we contribute to the field of gastric cancer pathological image classification by utilizing the GasHisSDB dataset. It has been explicitly designed to evaluate the effectiveness of shallow learning classifiers using both HC features and deep features derived from pre-trained Convolutional Neural Network (CNN) architectures and to investigate the extent to which these general features, without any specific fine-tuning strategy, can be effectively used in the medical context of gastric cancer classification.

The rationale for this approach is two-fold. First, by employing these deep features without any specific optimization or fine-tuning, we aim to assess their inherent potential, transferability, and general applicability of these features to medical imaging tasks. This investigation is important for understanding the robustness and effectiveness of general-purpose features in specialized domains, particularly when resources for fine-tuning or designing custom models may be limited.

Second, establishing a baseline performance using general features allows us to benchmark the potential gains that can be achieved through more specific adaptations in future work. This study serves as a foundational step, providing insights into the inherent capabilities of pre-trained models in the medical imaging domain, which can inform and guide subsequent efforts in fine-tuning and custom model design.

Moreover, we investigate feature fusion techniques, exploring the combination of both HC and deep features. This exploration allows us to examine how integrating different types of features can lead to improved classification performance. By understanding the synergies between these feature sets, we can identify optimal strategies for enhancing the robustness and accuracy of pathology image classification.

Additionally, we conducted a cross-magnification experiment to assess how varying image resolutions affect the performance of classification models in histopathological image analyses. This experiment is particularly important as it addresses the practical challenges faced in histopathology, where images may be captured at different magnifications [10,11]. High-magnification images provide detailed cellular structures, while low-magnification images offer a broader tissue architecture perspective [10,11]. By analyzing how different resolutions affect classification accuracy, we contribute valuable knowledge that can inform future practices in the field, ensuring that classifiers are effective and adaptable to the diverse conditions under which pathology images are obtained.

To sum up, the contributions of this work are the following:

- We proposed a comparative analysis of various HC and deep features across four different ML classifiers to identify the most stable and high-performing feature–classifier pairs for classifying gastric cancer histopathological images and distinguishing between normal and abnormal cells;
- We explored and analyzed various feature fusion techniques to determine their effectiveness in enhancing classification accuracy in the task at hand;
- We conducted a cross-magnification experiment to evaluate the impact of different image resolutions on classification performance, providing insights into the efficacy of utilizing multiple magnifications in pathology image analyses;
- Since different magnifications highlight unique tissue features, we conducted a cross-magnification experiment to assess the impact of varying image resolutions on classification performance, providing insights on the use of different magnifications in this field;
- We thoroughly evaluated the GasHisSDB dataset and compared our results with the state-of-the-art techniques.

The rest of this manuscript is organized as follows. Section 2 reviews the existing literature to contextualize our research within the field. Section 3 outlines the dataset and methodologies employed in this study. Section 4 presents the findings of our experiments, highlighting the performance of different feature categories, various classifiers, and feature combinations. Section 5 offers an in-depth analysis of our results, comparing them with previous studies and exploring their implications. Finally, the Section 6 summarizes our contributions and suggests directions for future research.

## 2. Related Work

The early detection and accurate diagnosis of gastric cancer (GC) is crucial, as patients with early-stage gastric cancer (EGC) have a much higher 5-year survival rate of 70–90% compared to only 10–30% for advanced gastric cancer (AGC) [9]. However, the accuracy of standard white-light endoscopy for detecting EGC is limited to 70–80%, heavily relying on the expertise of the endoscopist [9]. In recent years, researchers have increasingly explored the use of Computer Vision (CV) and DL techniques to assist in detecting and classifying gastric cancer from endoscopic and pathological images [12].

One of the first studies in this area was by Hirasawa et al., who developed a novel CNN for detecting and recognizing gastric cancer in video images [3]. Similarly, Yoon et al. developed an optimized CNN model for EGC detection and prediction [7]. Beyond endoscopic image analyses, researchers have also explored the use of CV techniques for gastric cancer classification using pathological images. For instance, Zhao et al. conducted a systematic review on the application of CNNs for identifying gastric cancer [4]. They found that a total of 27 studies had used CNN-based models for gastric cancer detection, classification, segmentation, and margin delineation from various medical imaging modalities, including endoscopy and pathology.

The reported accuracy of the CNN-based systems ranged from 77.3% to 98.7%, demonstrating the strong potential of these techniques for assisting clinicians in the diagnosis of gastric cancer [4]. One notable study in this domain was by Xie et al., who developed an optimized GoogleNet model for the diagnosis of gastric cancer pathological images [5]. Their improved model, which combined the strengths of two network structures, achieved a sensitivity of 97.61% and a specificity of 99.47% in recognizing gastric cancer pathological sections [5].

In this context, Hu et al. proposed a comprehensive dataset, named GasHisSDB, with 245,196 sub-sized gastric histopathology images labeled as normal or gastric cancer, which were derived from 600 whole slide images (WSIs) [2]. It was introduced to overcome the limitations of the existing datasets, particularly their small sample sizes [2,13]. Several follow-up studies have used the GasHisSDB dataset, starting from its proposal, where Hu et al. evaluated the performance of various ML and DL models [8], while several authors proposed optimized approaches to accomplish this task. For instance, Yong et al. proposed an ensemble DL approach based on EfficientNetB0, EfficientNetB1, DenseNet-121, DenseNet169, and MobileNet [6] whereas Li et al. introduced a lightweight gated fully fused network (LGFFN) with a gated hybrid input (GHI) module. The LGFFN-GHI comprises two main components: feature extraction and classification modules. The feature extraction module uses a cross-attention mechanism to fuse features from different scales. The classification module then takes the fused features and outputs the final classification prediction [14]. In addition, Fu et al. proposed MCLNet, a multidimensional CNN based on ShuffleNet. It extracts the correlation features between pixels in an image by one-dimensional convolution to achieve pixel-level and patch-level feature interaction.

Overall, the reviewed studies demonstrate significant progress in applying CV and DL techniques for gastric cancer classification from endoscopic and pathological images. However, despite these advances, there remain notable gaps and challenges. Many existing studies rely heavily on specific fine-tuning and optimization strategies tailored to particular datasets or clinical settings, which may limit the generalizability and transferability of their findings to broader contexts.

In this context, our study aims to advance the classification of gastric cancer using histopathologic images by addressing these gaps. Our primary objective is to propose a robust system that does not rely on ad hoc adjustments or fine-tuning. By leveraging features from non-optimized yet generic methods, we explore the feasibility of offering a generalizable solution that performs consistently across various magnifications and datasets. This approach is crucial for developing automated diagnostic tools that can be widely applicable and effective in diverse clinical environments.

Our study builds on the foundational work in this field by providing a comprehensive comparative analysis of both HC and deep features across multiple classifiers. We investigate the inherent potential and transferability of general features extracted from pre-trained CNNs without specific optimization. This analysis is important for understanding the robustness and effectiveness of general-purpose features in specialized domains, particularly when resources for fine-tuning or designing custom models may be limited.

Additionally, we explore feature fusion techniques to assess how integrating different types of features can enhance classification performance. By understanding the synergies between HC and deep features, we aim to identify optimal strategies for improving the robustness and accuracy of pathology image classification. Furthermore, we conduct cross-magnification experiments to evaluate the impact of different image resolutions on classification performance, addressing practical challenges faced in histopathology where images may be captured at varying magnifications.

In summary, our study contributes to the field by providing a detailed comparative analysis of feature extraction methods, exploring feature fusion strategies, and evaluating the effects of image magnification on classification accuracy. These efforts aim to develop a more generalizable and effective approach for automated gastric cancer diagnoses, advancing the application of ML and DL techniques in gastric cancer classification and addressing key limitations in current research that can influence the performance of the systems, such as the size and diversity of the training datasets, the specific CNN architectures employed, and the clinical context in which they are deployed.

## 3. Materials and Methods

This section provides details of the components used in our study. We begin with an overview of the dataset in Section 3.1, detailing its composition and relevance to our research objectives. Following this, in Section 3.2, we present both feature extraction methods employed, HC and deep, whereas in Section 3.3, we describe the classification methods applied. In addition, we discuss the performance evaluation measures in Section 3.4. Finally, Section 3.5 outlines the experimental setup and implementation details.

### 3.1. Dataset

**Figure 1:**
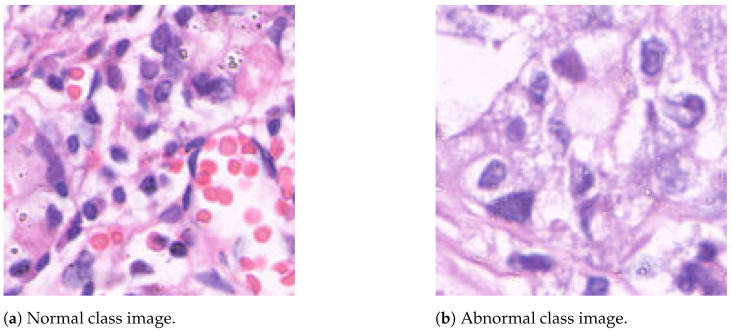
Sample images from the GasHisSDB dataset, acquired with the H&E staining method. The hematoxylin is alkaline, and stains cell nuclei a purplish blue, and eosin is acidic and stains the extracellular matrix and cytoplasm pink, with other structures taking on different shades, hues, and combinations of these colors [2].

The GasHisSDB dataset is a publicly available gastric histopathology image dataset [2]. It contains a total of 245,196 sub-sized gastric histopathology images, which were derived from 600 WSIs, stained with H&E, of 2048×2048 pixels. The images were scanned using a NewUsbCamera and digitized at 20× magnification. Two experienced pathologists from Liaoning Cancer Hospital and Institute provided the labels, classifying the images as either normal or abnormal (gastric cancer). A normal image is characterized by the absence of cancerous regions, reflecting typical microscopic cell observations. In contrast, an image is labeled as abnormal when approximately 50% of its area is occupied by cancerous regions [2]. The dataset is divided into three image sub-databases, each of them containing images with specific resolutions: 160×160 (S-A), 120×120 (S-B), and 80×80 (S-C) pixels. The distribution of the dataset images is summarized in Table 1, while Figure 1 shows two image samples.

### 3.2. Feature Extraction Methods

Features derived from images include a wide array of descriptors designed to capture morphological, pixel-level, and textural information, denoted as handcrafted features. As noted by [15], HC features can be broadly categorized into three main groups: invariant moments, texture features, and color features. To them, we have added a set of deep features, i.e., features obtained by the activations of off-the-shelf CNNs. In the following, we present a brief summary of each category along with the specific descriptors utilized.

#### 3.2.1. Invariant Moments

An image moment is a weighted average of pixel intensities in an image used to extract specific properties. Moments are crucial in image analyses and pattern recognition, helping to characterize segmented objects [16]. This study employs three distinct types of moments: Zernike, Legendre, and Chebyshev. A concise overview of these moment types follows.

**Chebyshev Moments (CH)** constitute a class of discrete orthogonal moments [17], based on Chebyshev polynomials [18] with the maximum possible leading coefficient constrained by an absolute value of 1 within the interval [−1,1]. This study used the first- and second-order moments, denoted as CH_1 and CH_2, respectively. Both moments were calculated to the fifth order. They are defined as

(1) $$T_{pq}=\sum_{x=0}^{M-1}\sum_{y=0}^{N-1}T_{p}\left(x\right)T_{q}\left(y\right)f\left(x,y\right)$$

where $T_{p}\left(x\right)$ and $T_{q}\left(y\right)$ are the Chebyshev polynomials of order *p* and *q*, respectively, and f(x,y) is the image function.

**Second-order Legendre Moments (LM)** are a type of continuous orthogonal moment that can be used for image analyses. LM capture information about the shape and orientation of an image. They are calculated using the second-order Legendre polynomials, which are orthogonal over the interval [−1,1] [19,20]; they can capture and represent objects’ shape and spatial characteristics within an image. In our analysis, we extracted the LM of order 5. The LM are defined as

(2) $$L_{pq}=\left(\frac{2p+1}{2}\right)\left(\frac{2q+1}{2}\right)\sum_{x=-1}^{1}\sum_{y=-1}^{1}P_{p}\left(x\right)P_{q}\left(y\right)f\left(x,y\right)$$

where $P_{p}\left(x\right)$ and $P_{q}\left(y\right)$ are the Legendre polynomials of order *p* and *q*, respectively, and f(x,y) is the image function.

**Zernike Moments (ZM)** are a type of continuous orthogonal moment that is defined over the unit circle [20]. They are calculated using Zernike polynomials, which form an orthogonal basis [21]. In this study, we extracted the ZM of order 5 with a repetition of 5. The ZM are defined as

(3) $$Z_{nm}=\frac{n+1}{\pi }\sum_{x=-1}^{1}\sum_{y=-1}^{1}V_{nm}^{*}\left(x,y\right)f\left(x,y\right)$$

where $V_{nm}\left(x,y\right)$ are the Zernike polynomials, $V_{nm}^{*}$ denotes the complex conjugate, *n* is the order, *m* is the repetition, and f(x,y) is the image function.

The specific forms of the Chebyshev, Legendre, and Zernike polynomials are provided below:

- **Chebyshev polynomial** $T_{p}\left(x\right)$ of order *p*:
  (4) $$T_{p}\left(x\right)=\cos\left(p\cdot \arccos\left(x\right)\right)$$
- **Legendre polynomial** $P_{p}\left(x\right)$ of order *p*:
  (5) Pp(x)=12p∑k=0p(−1)kpk2p−2kpxp−2k
- **Zernike polynomial** $V_{nm}\left(x,y\right)$ of order *n* with repetition *m*:
  (6) $$V_{nm}\left(x,y\right)=R_{nm}\left(\rho \right)\cdot e^{jm\theta }$$
  where $\rho =\sqrt{x^{2}+y^{2}}$ and θ=arctan(y/x), and $R_{nm}\left(\rho \right)$ is the radial polynomial defined as
  (7) $$R_{nm}\left(\rho \right)=\sum_{s=0}^{(n-|m|)/2}{\left(-1\right)}^{s}\frac{(n-s)!}{s!((n+|m|)/2-s)!((n-|m|)/2-s)!}\rho^{n-2s}$$

#### 3.2.2. Texture Features

Texture serves as a visual feature indicative of homogeneity within an image. It reveals the organization and arrangement of surface structures exhibiting gradual or periodic variations. Rather than relying on individual pixel characteristics, a texture analysis requires statistical calculations over regions encompassing multiple pixels [22]. The texture is characterized by the gray-level distribution of pixels and their surrounding spatial neighbors, encapsulating local texture information. Additionally, global texture information is determined by the extent of repetition of this local texture information. For the sake of this work, we have considered two widely employed methods, now described.

**Rotation-Invariant Haralick (HAR) Features**: Thirteen HAR features were extracted from the Gray-Level Co-occurrence Matrix (GLCM) and then transformed into rotation-invariant features [23]. To achieve rotation invariance, four variations of the GLCM were calculated, each with a distance parameter d=1 and angular orientations $\theta =[0^{\circ },45^{\circ },90^{\circ },135^{\circ }]$.

The Gray-Level Co-occurrence Matrix (GLCM) is defined as

(8) $$P\left(i,j,d,\theta \right)=\sum_{x=1}^{N}\sum_{y=1}^{N}\left\{\begin{array}{cc} 1 & \mathrm{if}\;I(x,y)=i\;\mathrm{and}\;I(x+d\cos\theta ,y+d\sin\theta )=j\\ 0 & \mathrm{otherwise} \end{array}\right.$$

From the GLCM, we extracted the first 13 HAR features. They are defined as follows:

(9) $$\mathrm{Angular}\;\mathrm{Second}\;\mathrm{Moment}\;\left(\mathrm{ASM}\right)=\sum_{i=0}^{N-1}\sum_{j=0}^{N-1}P{\left(i,j\right)}^{2}$$

(10) $$\mathrm{Contrast}=\sum_{n=0}^{N-1}n^{2}\left(\sum_{i=0}^{N-1}\sum_{j=0}^{N-1}P\left(i,j\right),\left|i-j\right|=n\right)$$

(11) $$\mathrm{Correlation}=\frac{\sum_{i=0}^{N-1}\sum_{j=0}^{N-1}\left(i\cdot j\cdot P\left(i,j\right)\right)-\mu_{x}\cdot \mu_{y}}{\sigma_{x}\cdot \sigma_{y}}$$

where

$$\begin{array}{cc} \mu_{x} & =\sum_{i=0}^{N-1}i\left(\sum_{j=0}^{N-1}P\left(i,j\right)\right)\\ \mu_{y} & =\sum_{j=0}^{N-1}j\left(\sum_{i=0}^{N-1}P\left(i,j\right)\right)\\ \sigma_{x} & =\sqrt{\sum_{i=0}^{N-1}{\left(i-\mu_{x}\right)}^{2}\left(\sum_{j=0}^{N-1}P\left(i,j\right)\right)}\\ \sigma_{y} & =\sqrt{\sum_{j=0}^{N-1}{\left(j-\mu_{y}\right)}^{2}\left(\sum_{i=0}^{N-1}P\left(i,j\right)\right)} \end{array}$$

(12) $$\mathrm{Variance}=\sum_{i=0}^{N-1}\sum_{j=0}^{N-1}{\left(i-\mu \right)}^{2}P\left(i,j\right)$$

where

(13) $$\mu =\sum_{i=0}^{N-1}\sum_{j=0}^{N-1}i\cdot P\left(i,j\right)$$

(14) $$\mathrm{Inverse}\;\mathrm{Difference}\;\mathrm{Moment}\;\left(\mathrm{IDM}\right)=\sum_{i=0}^{N-1}\sum_{j=0}^{N-1}\frac{P(i,j)}{1+{\left(i-j\right)}^{2}}$$

(15) $$\mathrm{Sum}\;\mathrm{Average}=\sum_{i=2}^{2N}i\cdot P_{x+y}\left(i\right)$$

where

(16) $$P_{x+y}\left(k\right)=\sum_{i=0}^{N-1}\sum_{j=0}^{N-1}P\left(i,j\right),\;i+j=k$$

(17) $$\mathrm{Sum}\;\mathrm{Variance}=\sum_{i=2}^{2N}{\left(i-\mathrm{Sum}\;\mathrm{Average}\right)}^{2}\cdot P_{x+y}\left(i\right)$$

(18) $$\mathrm{Sum}\;\mathrm{Entropy}=-\sum_{i=2}^{2N}P_{x+y}\left(i\right)\log P_{x+y}\left(i\right)$$

(19) $$\mathrm{Entropy}=-\sum_{i=0}^{N-1}\sum_{j=0}^{N-1}P\left(i,j\right)\log P\left(i,j\right)$$

(20) $$\mathrm{Difference}\;\mathrm{Variance}=\mathrm{Variance}\;\mathrm{of}\;P_{x-y}\left(k\right)$$

where

(21) $$P_{x-y}\left(k\right)=\sum_{i=0}^{N-1}\sum_{j=0}^{N-1}P\left(i,j\right),\;\left|i-j\right|=k$$

(22) $$\mathrm{Difference}\;\mathrm{Entropy}=-\sum_{i=0}^{N-1}P_{x-y}\left(i\right)\log P_{x-y}\left(i\right)$$

(23) $$\mathrm{Information}\;\mathrm{Measures}\;\mathrm{of}\;\mathrm{Correlation}\;1=\frac{HXY-HXY1}{\max\{HX,HY\}}$$

where

$$\begin{array}{cc} HXY & =-\sum_{i=0}^{N-1}\sum_{j=0}^{N-1}P\left(i,j\right)\log P\left(i,j\right)\\ HXY1 & =-\sum_{i=0}^{N-1}\sum_{j=0}^{N-1}P\left(i,j\right)\log\left(P_{x}\left(i\right)P_{y}\left(j\right)\right)\\ HX & =-\sum_{i=0}^{N-1}P_{x}\left(i\right)\log P_{x}\left(i\right)\\ HY & =-\sum_{j=0}^{N-1}P_{y}\left(j\right)\log P_{y}\left(j\right) \end{array}$$

(24) $$\mathrm{Information}\;\mathrm{Measures}\;\mathrm{of}\;\mathrm{Correlation}\;2=\sqrt{1-\exp\left(-2(HXY2-HXY)\right)}$$

where

(25) $$HXY2=-\sum_{i=0}^{N-1}\sum_{j=0}^{N-1}P_{x}\left(i\right)P_{y}\left(j\right)\log\left(P_{x}\left(i\right)P_{y}\left(j\right)\right)$$

**Local Binary Pattern (LBP)** is a powerful method for capturing the texture and patterns in an image, as described in [24]. In this study, we computed the histogram of the LBP and transformed it into a rotation-invariant form [25]. This histogram was then extracted and used as the feature vector. The LBP map was generated within a neighborhood defined by a radius of r=1 and eight neighbors (n=8).

The LBP operator assigns a binary code to each pixel by thresholding its neighborhood with the center pixel value. The LBP code for a pixel $\left(x_{c},y_{c}\right)$ is given by

(26) $$LBP_{P,R}=\sum_{p=0}^{P-1}s\left(i_{p}-i_{c}\right)2^{p}$$

where s(x) is the sign function

(27) $$s\left(x\right)=\left\{\begin{array}{cc} 1 & \mathrm{if}\;x\geq 0\\ 0 & \mathrm{otherwise} \end{array}\right.$$

#### 3.2.3. Color Features

Histograms are the most widely employed method for characterizing the color properties of images since they effectively represent the global color distribution within an image, indicating their proportion. The descriptors that can be extracted from the histogram are invariant to image rotation, translation, and scaling changes. However, they have a significant limitation in that they cannot describe the local distribution of colors, the spatial location of each color, or specific objects within the image [26]. In this study, these descriptors were calculated from images that underwent a conversion to grayscale, streamlining the process of the analysis and computation.

**Histogram (Hist) Features:** From the histogram, which characterizes the overall color distribution within the image, we derived seven statistical descriptors: the mean, standard deviation, smoothness, skewness, kurtosis, uniformity, and entropy.

For a histogram h(i) with *N* bins, the statistical descriptors are defined as follows:

(28) $$\begin{array}{cc} \mathrm{Mean} & =\frac{1}{N}\sum_{i=0}^{N-1}i\cdot h\left(i\right) \end{array}$$

(29) $$\begin{array}{cc} \mathrm{Standard}\;\mathrm{Deviation} & =\sqrt{\frac{1}{N}\sum_{i=0}^{N-1}{\left(i-\mu \right)}^{2}\cdot h\left(i\right)} \end{array}$$

(30) $$\begin{array}{cc} \mathrm{Smoothness} & =1-\frac{1}{1+\mathrm{Variance}} \end{array}$$

(31) $$\begin{array}{cc} \mathrm{Skewness} & =\frac{1}{N\sigma^{3}}\sum_{i=0}^{N-1}{\left(i-\mu \right)}^{3}\cdot h\left(i\right) \end{array}$$

(32) $$\begin{array}{cc} \mathrm{Kurtosis} & =\frac{1}{N\sigma^{4}}\sum_{i=0}^{N-1}{\left(i-\mu \right)}^{4}\cdot h\left(i\right)-3 \end{array}$$

(33) $$\begin{array}{cc} \mathrm{Uniformity} & =\sum_{i=0}^{N-1}h{\left(i\right)}^{2} \end{array}$$

(34) $$\begin{array}{cc} \mathrm{Entropy} & =-\sum_{i=0}^{N-1}h\left(i\right)\log h\left(i\right) \end{array}$$

**Autocorrelogram (AC):** The AC captures the spatial correlation of colors within an image. It is a restricted version of the more general color correlogram, considering only the spatial correlation between pixels of the same color [27]. Specifically, the color autocorrelogram calculates the probability that a pixel of a given color will be found at a certain distance, *d*, away from another pixel of the same color. Our research considered four discrete distances: d=1,2,3,4. The four resulting probability vectors are concatenated to form a comprehensive feature vector.

The autocorrelogram for color *k* at distance *d* is defined as

(35) $${\mathrm{AC}}_{k}\left(d\right)=\frac{1}{N_{k}}\sum_{i=1}^{N_{k}}\sum_{j\in N_{d}\left(i\right)}\delta \left(C\left(i\right),k\right)\delta \left(C\left(j\right),k\right)$$

where $N_{k}$ is the number of pixels of color *k*, $N_{d}\left(i\right)$ is the set of pixels at distance *d* from pixel *i*, and δ is the Kronecker delta function.

**Haar-like (Haar) Features**: The key idea behind these features is to calculate the difference in the sum of pixel intensities across rectangular regions in an image. This allows detecting edges, lines, and center-surround features that indicate the presence of an object [28]. Haar-like features can be calculated using integral images to speed up the process. The integral image at a location (x,y) is defined as

(36) $$II\left(x,y\right)=\sum_{x^{\prime }\leq x}\sum_{y^{\prime }\leq y}I\left(x^{\prime },y^{\prime }\right)$$

Using the integral image, the sum of pixel intensities within a rectangular region can be computed efficiently, allowing for the calculation of Haar-like features.

#### 3.2.4. Deep Features

With deep features, we refer to the characteristics of an image derived from the CNN activations since they have proven to be a potent strategy for enhancing the predictive power of classifiers [29]. These deep features were extracted from off-the-shelf CNN architectures pre-trained on the well-known natural image dataset ImageNet [30].

Specifically, depending on the architecture, deep features were extracted from one of the following layers: (i) the penultimate layer, (ii) the final fully connected layer, or (iii) the last pooling layer. This approach ensures extracting features encapsulating the network’s learned global knowledge. Notably, the fine-tuning strategy for the classification phase was not employed to maintain the generalization ability of the networks [31,32]. Detailed specifications regarding the selected layers for feature extraction, input dimensions, and the count of trainable parameters for each CNN model are outlined in Table 2, while a brief explanation of the CNNs employed is now provided.

**AlexNet** consists of a sequence of convolutional and max-pooling layers, culminating in three fully connected layers [33]. With only five convolutional layers, it represents the most shallow architecture used in this study.

**DarkNet** builds upon the established principles of inception and batch normalization. This study employs two specific versions of DarkNet, incorporating 19 [34] and 53 [35] convolutional layers. These configurations form the foundational network for the You Only Look Once object detection method.

**DenseNet**, proposed by Huang et al. [36], addresses the typical CNN characteristic of having layers equal to the number of connections. Specifically, the number of connections is L(L+1)/2, where *L* denotes the number of layers. Each layer’s input comprises the output from all preceding layers, which then serves as the input for the subsequent layer.

**EfficientNet** stands out for its uniform and efficient scaling of network width, depth, and resolution through compound scaling. Proposed by Tan et al. [37], this study employs the EfficientNetB0 version.

**Inception-v3** uses the inception layer concept by incorporating factorized, smaller, and asymmetric convolutions [38]. Inception models are notable for their multi-branch architectures, combining filters of various sizes integrated through concatenation within each branch.

**Inception-ResNet-v2** merges the strengths of ResNet and Inception architectures [39]. The Inception-ResNet block combines variously sized convolutional filters with residual connections, featuring four max-pooling layers and 160 convolutional layers.

**ResNet** refers to a family of deep architectures that use residual learning [40]. These architectures integrate skip-connections or recurrent units to link blocks of convolutional and pooling layers, with each block followed by batch normalization [43]. This study employs three ResNet variants, ResNet-18, ResNet-50, and ResNet-101, with the numbers indicating the respective network depths.

**VGG** comprises a series of convolutional layers followed by max-pooling, which enhances its deep representation capabilities [41]. This study uses VGG19, featuring 19 layers.

**XceptionNet** extends the Inception architecture by employing depth-wise separable convolutions to improve efficiency and reduce parameter count. This approach aims to capture complex feature dependencies by focusing on cross-channel correlations [42].

### 3.3. Classification Methods

After feature extraction, HC and deep features served as inputs for four classical ML algorithms to classify GasHisSDB. Here is a brief overview of these classifiers.

**Decision Tree (DT)** is a hierarchical data structure used for prediction. Each internal node represents a feature, with branches denoting possible feature values and leaves representing different categories. The algorithm optimizes this structure by pruning nodes that minimally contribute to category separation, thereby merging instances at higher levels. Classification is achieved by tracing the path from the root to a leaf node [44].

**k-Nearest Neighbor (kNN)**: The kNN classifier categorizes observations by considering the classes of the k training examples nearest to the observation in question. This method employs a local strategy for classification, leveraging the proximity of neighboring instances to determine the class [45].

**Support Vector Machine (SVM)**: SVM differentiates categories by mapping examples to opposite sides of a decision boundary. The one-vs.-rest approach is employed for multiclass problems, training individual classifiers to distinguish each class from all others [46].

**Random Forest (RF)**: This algorithm aggregates predictions from multiple Decision Trees, each constructed from random subsets of features and examples. By fostering diversity among the trees, this ensemble method enhances model robustness, improving resilience against data imbalance and mitigating overfitting. The use of 100 trees specifically enhances the random forest’s predictive accuracy [47].

### 3.4. Performance Evaluation Measures

In evaluating the performance of a binary classifier on a dataset, each instance is classified as either negative or positive based on the classifier’s predictions. The result of this classification, when compared to the actual target value, determines the following performance measures:

- **True Negatives (TNs)**: instances correctly predicted as negative.
- **False Positives (FPs)**: instances incorrectly predicted as positive.
- **False Negatives (FNs)**: instances incorrectly predicted as negative.
- **True Positives (TPs)**: instances correctly predicted as positive.

As detailed below, we assess the classifier’s performance using several measures specifically defined for binary classification tasks.

- **Accuracy (A)**: It is the ratio of correct predictions to the total number of predictions:
  $$A=\frac{TP+TN}{TP+FN+FP+TN}$$
- **Precision (P)** is the ratio of *TP*s to the sum of *TP*s and *FP*s, indicating the classifier’s efficiency in predicting positive instances:
  $$P=\frac{TP}{TP+FP}$$
- **Recall (R)**, also known as sensitivity, is the ratio of *TP*s to the sum of *TP*s and *FN*s:
  $$R=\frac{TP}{TP+FN}$$
- **Specificity (S)** is the ratio of *TN*s to the sum of *TN*s and *FP*s:
  $$S=\frac{TN}{TN+FP}$$
- **F1-score (F1)** is the harmonic mean of P and R, considering both *FP*s and *FN*s:
  $$F1=2\cdot \frac{P\cdot R}{P+R}$$
- **Matthews Correlation Coefficient (MCC)** is a comprehensive measure that considers all elements of the confusion matrix (TP, TN, FP, FN). Ranging from −1 to +1, it provides a high score only when the classifier performs well in both the positive and negative classes:
  $$MCC=\frac{TP\cdot TN-FP\cdot FN}{\sqrt{\left(TP+FP)(TP+FN)(TN+FP)(TN+FN\right)}}$$
- **Balanced accuracy (BACC)** is defined as the mean of specificity and sensitivity:
  $$BACC=\frac{S+R}{2}$$

### 3.5. Experimental Setup

The experiments were performed on a workstation with an Intel(R) Core(TM) i9-8950HK @ 2.90 GHz CPU, 32 GB of RAM, and an NVIDIA GTX1050 Ti GPU with 4 GB of memory. MATLAB R2021b was used for all implementations and experimental evaluations.

This study deliberately did not use image augmentations to concentrate on extracting pure features from the original images. Moreover, we used Euclidean distance as a distance measure for kNN with k=1; note that with k=1, no voting strategy is required. In addition, the SVM kernel function uses a linear kernel, and the number of DTs composing the RF has been set to 100.

In addition, a 5-fold cross-validation (CVal) approach was employed for the testing strategy. This method ensures statistical reliability by repeatedly training and testing the same dataset. Specifically, the dataset is divided into 80% for training and 20% for testing at each iteration.

## 4. Experimental Results

In this section, we detail the comprehensive experimental analysis conducted to evaluate the performance of various feature extraction and classification techniques on the GasHisSDB dataset. Section 4.1 presents the results obtained with HC features, whereas Section 4.2 explores the use of CNN as feature extractors. This is succeeded by Section 4.3, where we discuss the outcomes of combining HC and deep features to enhance classification accuracy. To ensure robustness across different magnifications, Section 4.4 evaluates the consistency and reliability of our methods when applied to images at various magnification levels. Finally, Section 4.5 provides a critical analysis of our results in the context of existing research. Finally, please note that for the sake of comparison, we have reported only the results obtained with the two best-performing classifiers, i.e., RF and SVM. We report kNN and DT results in Appendix A.

### 4.1. HC Feature Performance

The outcomes obtained by RF and SVM trained with HC features are presented in Table 3 and Table 4, while Table A1 and Table A2 report the results obtained with DT and kNN, respectively.

The performance obtained with the SVM (Table 3) shows that the CH_1 and CH_2 features provide the best accuracy (75.92% and 72.50%, respectively) and balanced accuracy (73.45% and 67.66%, respectively), showing their potential for discriminating between normal and abnormal tissues. Interestingly, while the Hist features achieve high precision (96.29%), it suffers from very low recall (9.00%), leading to a much lower balanced accuracy (54.23%).

As for the RF (Table 4), when trained with the LBP features, it achieves the highest accuracy (79.57%), precision (80.74%), and F1 (83.77%). The LBP feature’s strong performance across most metrics suggests its effectiveness in capturing essential patterns in histopathological images. Contrastively, the Haar feature again demonstrates a significantly lower accuracy (62.48%) and balanced accuracy (53.97%), indicating its relative ineffectiveness in this context.

Finally, the LBP again emerges as the top performer with both DT (Table A1) and kNN (Table A2). DT with LBP obtained an accuracy of 71.22% and a BACC of 69.87%. This indicates that despite the DT classifier’s simplicity, LBP features can still capture discriminative information effectively. Haar features, however, perform poorly with a balanced accuracy of 53.37%. Instead, kNN with LBP obtained an accuracy of 69.51% and an F1 of 74.58%. Conversely, the Haar feature shows poor performance with an accuracy of 42.17% and a balanced accuracy of 48.53%, reaffirming its limitations for this task.

The consistency across classifiers underlines LBP features’ robustness, even without top-notch performance.

### 4.2. Deep Feature Performance

The results of SVM and RF classifiers trained using deep features are summarized in Table 5 and Table 6. In Table A3 and Table A4, the results obtained with DT and kNN are reported, respectively.

As shown in Table 5, SVM trained with DenseNet-201 features achieves the highest accuracy (86.02%) and balanced accuracy (84.23%), followed closely by DarkNet-53 and EfficientNetB0.

Even with RF (Table 6), DenseNet-201 achieves the highest accuracy (91.93%) and balanced accuracy (91.33%), indicating its superior feature extraction capability. Other deep features, such as those from DarkNet-53 and ResNet-101, also perform exceptionally well with RF.

In addition, the features extracted from DenseNet-201 also excel with DT and kNN. As shown in Table 6, DT achieves the highest accuracy (84.92%) and balanced accuracy (84.20%). In contrast, kNN (Table A4) shows superior performance with DenseNet-201 and DarkNet-53, which gained accuracies of 88.21% and 88.25%, respectively, and balanced accuracies of 87.23% and 87.22%. These results further confirm that even simpler classifiers can benefit significantly from the rich feature representations pre-trained CNNs provide.

This consistent top performance across different classifiers highlights DenseNet-201’s strong feature extraction capabilities for histopathological images, even without any fine-tuning strategy.

### 4.3. Feature Fusion Performance

Despite the significant results achieved from the previous classification, further efforts were made to enhance performance. An additional experiment explored the potential of integrating the representative power of both HC and deep features into a feature fusion strategy. More specifically, this experiment focused on LBP, DenseNet-201, and EfficientNetB0, which resulted in the best HC and the best two deep features, respectively. They were evaluated with all possible combinations using the best three classifiers from the previous stage: DT, SVM, and RF. This integration aimed to leverage their combined strengths for improved performance. The results are shown in Table 7.

For the fusion of LBP and DenseNet-201 features, SVM emerged as the most effective classifier with an accuracy of 94.41% and F1 of 95.40%. This performance indicates that SVM, when trained with this fusion of features, is highly reliable. RF also performed robustly with an accuracy of 92.16%, showing strong capability but slightly lagging behind SVM.

In the case of combining LBP with EfficientNetB0 features, SVM again demonstrated superior performance with an accuracy of 94.05%, F1 of 95.11%, MCC of 87.53, and balanced accuracy of 93.68%. This reiterates SVM’s effectiveness across different feature combinations. RF showed a notable drop in performance compared to the previous fusion strategy, suggesting that this combination might not be as effective for RF.

The combination of DenseNet-201 and EfficientNetB0 features led to SVM achieving the highest measures overall, with an accuracy of 94.89% and F1-score of 95.78%. This indicates that the deep features from these two CNNs complement each other well, providing rich information for the classifier. RF and DT also performed better with this fusion strategy than HC features, highlighting the benefit of purely deep features.

Finally, the most complex feature fusion strategy, combining LBP with both DenseNet-201 and EfficientNetB0, resulted in the highest overall performance for SVM, with an accuracy of 95.03%, and F1 of 95.90%. This indicates that the incorporation of both HC and multiple deep features provides a comprehensive feature set that enhances classification performance. RF also showed its best performance with this fusion strategy, suggesting that adding more feature types helps improve its robustness and generalization.

### 4.4. Cross-Magnification Performance

Table 8 and Table 9 detail the performance measures of two cross-magnification experiments conducted using different classifiers and feature fusion strategies on the GasHisSDB dataset. The experiments involved training on a 160×160 image sub-database and testing on smaller dimensions (120×120 and 80×80, respectively).

**Results of testing on S-B:** The classifiers were evaluated on the 120×120 test set in the first experiment. Combining LBP and DenseNet-201 as features yielded varied results across different classifiers. The RF classifier outperformed others, achieving an accuracy of 89.04%, an F1 of 90.76%, and a balanced accuracy of 89.76%. The SVM also demonstrated strong performance, particularly with a precision of 97.42%, though it lagged in recall compared to RF.

When integrating LBP with EfficientNetB0, the performance metrics showed a slight decline, especially noticeable in the DT classifier, which recorded an accuracy of 85.17%. The RF continued to maintain relatively high performance, albeit slightly lower than with DenseNet-201.

The fusion of DenseNet-201 and EfficientNetB0 features displayed a notable improvement in classifier performance. RF again led the results with an accuracy of 89.55%, an F1-score of 91.30%, and a balanced accuracy of 89.90%. The DT classifier also performed well under this strategy, achieving high precision and recall rates.

Combining all three feature sets (LBP, DenseNet-201, and EfficientNetB0) resulted in marginal improvements across the board. RF achieved the highest accuracy at 89.56%, while the SVM exhibited the highest precision at 98.45%. This comprehensive feature fusion strategy enhanced the robustness and consistency of the classifiers’ performance, particularly evident in the balanced accuracy and MCC scores.

**Results of testing on S-C:** The second experiment, with testing on the 80×80 sub-database, illustrated a greater challenge for the classifiers, reflected in the generally lower performance values. The LBP + DenseNet-201 combination showed that RF remained the most reliable classifier with an accuracy of 78.89% and an F1-score of 79.73%. While demonstrating high precision at 96.18%, the SVM struggled with recall and balanced accuracy, indicating a possible reliance on the 160×160 pixels’ image data.

In the LBP + EfficientNetB0 strategy, all classifiers showed decreased performance, with the SVM particularly underperforming in terms of recall and F1. RF again stood out, albeit with lower scores than the previous experiment.

The fusion of DenseNet-201 and EfficientNetB0 improved the values slightly, with RF achieving an accuracy of 79.73% and a balanced accuracy of 81.69%. This strategy illustrated a more balanced performance across the classifiers, with DT and SVM showing moderate improvements in precision and recall.

Lastly, the combination of all three feature sets in this second experiment underscored RF as the most robust classifier with an accuracy of 78.44% and an F1 of 79.09%. The SVM showed better balanced accuracy compared to previous setups, though it still struggled with recall.

### 4.5. Comparison with the State of the Art

Table 10 showcases a comparative analysis of the performance of our work against previous state-of-the-art studies on the GasHisSDB dataset.

In Hu et al.’s work [2], two models, VGG16 and ResNet50, were tested with a 40/40/20 split. VGG16 achieved accuracies of 96.12%, 96.47%, and 95.90% across S-C, S-B, and S-A, respectively. Similarly, ResNet50 showed comparable performance with 96.09%, 95.94%, and 96.09% in the same sub-databases.

In the study of [48], an InceptionV3 model trained from scratch using a 40/20/40 split achieved a remarkable 98.83% accuracy in the S-A sub-database. Furthermore, combining InceptionV3 and ResNet50 through feature concatenation yielded a very close accuracy of 98.80%.

Li et al. [14] used a local–global feature fuse network (LGFFN) with a 60/20/20 split, achieving an accuracy of 96.81% in the S-A. This approach leverages the strengths of local and global features to improve classification performance. On the other hand, [49] employed MCLNet based on ShuffleNetV2 with an 80/-/20 split, reporting high accuracies of 96.28%, 97.95%, and 97.85% across S-C, S-B, and S-A, respectively.

The ensemble method adopted in [6] with a 40/20/40 split exhibited outstanding results, with accuracies of 97.72%, 98.68%, and 99.20% in S-C, S-B, and S-A, respectively. This ensemble approach amalgamates the strengths of multiple models, thereby achieving superior performance and robustness in classification tasks.

The main differences between the state of the art and our work are that our evaluation followed a 5-fold Cval protocol; we used only HC features and features extracted from pre-trained, off-the-shelf CNNs to evaluate the extent to which non-specialized and non-tuned features can accomplish the binary classification task faced in this study. We tested on S-B and S-C by using only models trained on S-A to investigate the influence of the image resolution size in this scenario.

As can be seen, we reported two models: SVM with feature fusion and RF with feature fusion. The SVM model achieved accuracies of 60.31%, 85.82%, and 95.03% in S-C, S-B, and S-A, respectively. Similarly, the RF model showed accuracies of 78.44%, 89.56%, and 92.26% in the same categories. Although lower in S-B and S-C due to the training/testing strategy employed, compared to previous studies, these results highlight the potential of feature fusion techniques in improving classification performance, even without the need for a complex fine-tuning strategy that can be time-consuming and, above all, require a high amount of labeled data that can be complex in medical scenarios [50]. The 5-fold CV method ensures a more robust evaluation by repeatedly training and testing on different subsets of the data, thus providing a reliable estimate of the models’ performance.

## 5. Discussion

This section analyzes the key aspects of our study. Specifically, Section 5.1 examines the relative performance and merits of HC versus deep features, whereas Section 5.2 discusses the outcomes of combining HC and deep features, analyzing how this fusion impacts the overall classification performance. Next, Section 5.3 evaluates the robustness and adaptability of our classification models across different magnifications of the considered dataset. Finally, Section 5.4 addresses the constraints and potential weaknesses of our study.

### 5.1. On the HC vs. Deep Feature Comparison

The comparative analysis presented in Section 4.1 and Section 4.2 reveals that, on the one hand, LBP consistently performs well among HC features, demonstrating robustness and reliability across different classifiers, even without exceptional performance. On the contrary, Haar features generally perform poorly, suggesting that they are less suitable for this task.

On the other hand, deep features extracted from pre-trained CNNs, especially DenseNet-201 and DarkNet-53, consistently outperform HC features. This underscores the advantage of using Deep Learning models for feature extraction in complex tasks such as histopathological image classification, even without fine-tuning strategies.

In addition, the random forest classifier has shown strong performance with both HC and deep features, indicating its versatility and effectiveness in handling various feature types. More precisely, RF with features extracted from DenseNet-201 demonstrated their reliability for the task.

In summary, the detailed performance evaluation across various feature–classifier combinations provides valuable insights into the strengths and weaknesses of different approaches. The consistent superiority of deep features, particularly those from DenseNet-201 and EfficientNetB0, suggests a clear direction in this domain, emphasizing the integration of advanced Deep Learning techniques for enhanced classification accuracy, effectiveness, and robustness. This is the main reason that motivated us to pick them along with LBP among the HC features to investigate feature fusion strategies.

### 5.2. On the Feature Fusion Performance

Across all feature fusion strategies, SVM consistently outperformed both DT and RF, demonstrating its superior ability to handle the diverse and complex feature sets derived from combining HC and deep features. SVM’s consistently high performance across various combinations suggests that it is highly adaptable to different types of features.

While generally strong, RF showed variability in performance depending on the feature combination, indicating that it might be more sensitive to the quality and type of features used. DT, on the other hand, consistently lagged behind SVM and RF, pointing to its relatively lower ability to utilize complex feature sets effectively.

The combination of LBP, DenseNet-201, and EfficientNetB0 features, mainly when used with SVM, provides the most reliable and high-performing strategy for classifying histopathological images. This fusion strategy leverages the strengths of both HC and deep features, resulting in a robust classification framework.

### 5.3. On the Cross-Magnification Performance

Overall, the experiments reveal that the RF classifier consistently outperforms DT and SVM across various feature fusion strategies and test set dimensions. The combination of DenseNet-201 and EfficientNetB0 generally provides the most reliable feature set, enhancing classifier performance and demonstrating the feasibility of using features provided by HC methods or pre-trained CNNs in histopathological image classification.

The comprehensive analysis demonstrates the efficacy of feature fusion and the importance of choosing robust classifiers to achieve high accuracy and reliability in medical image classification tasks.

### 5.4. Limitations

While comprehensive in its approach to evaluating the performance of shallow learning classifiers on histopathological image classification, this study presents several limitations that must be acknowledged.

First, reliance on pre-trained CNNs for feature extraction without any fine-tuning specific to the dataset at hand may limit the potential performance of the classifiers. Fine-tuning these networks could potentially yield features more tailored to the specific characteristics of the histopathological images, thereby improving classification accuracy.

Second, the experiments were conducted using a single dataset, GasHisSDB, which might limit the generalizability of the findings. The performance measures observed might vary significantly when applied to other histopathological datasets with different image characteristics, variations in staining procedures, or differing disease profiles. A broader validation across multiple datasets would provide more robust evidence of the classifiers’ effectiveness.

Third, this study employed a specific image resolution sub-database (160×160 for training and testing, 120×120 and 80×80 for testing). The impact of image resolution on classifier performance was not extensively explored, and it is possible that different resolutions could influence the feature extraction and classification processes.

Additionally, this study did not incorporate image augmentation techniques commonly used in image classification tasks to improve model generalization by artificially increasing the size and variability of the training dataset. The absence of augmentation may result in overfitting, particularly given the limited data available for training.

In summary, while this work provides valuable insights into the use of shallow learning classifiers for histopathological image classification with features supplied by non-fine-tuned methods, the limitations discussed here suggest avenues for further research to enhance and validate the findings.

## 6. Conclusions

This study comprehensively evaluates shallow learning classifiers for histopathological image classification using HC and deep features.

The comparative analysis of HC versus deep features demonstrates the clear superiority of deep features, particularly those extracted from pre-trained CNNs such as DenseNet-201 and EfficientNetB0. These features consistently outperform HC features, highlighting the advanced feature extraction capability of DL models in complex image classification tasks. Among HC features, LBP shows robust performance, while Haar features are less effective.

Our exploration of feature fusion techniques shows that combining features can significantly enhance classification performance. The SVM classifier, in particular, excels in handling diverse and complex feature sets, outperforming both DT and RF classifiers across various combinations. The fusion of LBP, DenseNet-201, and EfficientNetB0 features emerges as the most reliable strategy, leveraging the strengths of both HC and deep features.

In addition, our cross-magnification experiments underscore the robustness of RF classifiers, which consistently perform well across different image resolutions. The combination of features from DenseNet-201 and EfficientNetB0 proves effective in maintaining high classification accuracy, demonstrating the feasibility of utilizing features from pre-trained CNNs and HC methods.

While this study advances our understanding of shallow learning classifiers in histopathological image classification, our results open the field for several future works. For instance, with feature importance and explainability techniques, we plan to investigate the most effective features for the classifiers’ final prediction to simplify the workflow with feature selection strategies. Moreover, we aim to integrate further DL methods like Vision Transformer and leverage them as feature extractors in this context.

## Figures and Tables

**Table 1:** Description of GasHisSDB with details on its subdivision and number of images per class.

Sub-Database	Size	Abnormal	Normal
S-A	160×160 pixels	13,124	20,160
S-B	120×120 pixels	24,801	40,460
S-C	80×80 pixels	59,151	87,500
Total		97,076	148,120

**Table 2:** Employed CNN details including reference paper, number of trainable parameters in millions, input shape, feature extraction layer, and related feature vector size.

CNN	Parameters (M)	Input Shape	Feature Layer	# of Features
AlexNet [33]	60	224×224	Pen. FC	4096
DarkNet-19 [34]	20.8	224×224	Conv19	1000
DarkNet-53 [35]	20.8	224×224	Conv53	1000
DenseNet-201 [36]	25.6	224×224	Avg. Pool	1920
EfficientNetB0 [37]	5.3	224×224	Avg. Pool	1280
Inception-v3 [38]	21.8	299×299	Last FC	1000
Inception-ResNet-v2 [39]	55	299×299	Avg. Pool	1536
ResNet-18 [40]	11.7	224×224	Pool5	512
ResNet-50 [40]	26	224×224	Avg. Pool	1024
ResNet-101 [40]	44.6	224×224	Pool5	1024
VGG19 [41]	144	224×224	Pen. FC	4096
XceptionNet [42]	22.9	299×299	Avg. Pool	2048

**Table 3:** Performance obtained with SVM trained with HC features. Values are shown in terms of %.

Descriptor	A	P	R	S	F1	MCC	BACC
AC	67.30	69.18	82.99	43.20	75.45	28.71	63.09
Haar	62.18	62.38	94.59	12.38	75.18	12.45	53.49
Hist	44.67	96.29	9.00	99.47	16.47	17.91	54.23
HAR	62.07	73.64	58.21	68.00	65.02	25.64	63.10
LBP	62.64	70.00	67.06	55.85	68.50	22.69	61.46
CH_1	75.92	77.37	85.14	61.75	81.07	48.61	73.45
CH_2	72.50	71.57	90.55	44.76	79.95	40.78	67.66
LM	73.32	72.65	89.73	48.11	80.29	42.61	68.92
ZM	63.84	69.78	71.08	52.72	70.43	23.93	61.90

**Table 4:** Performance obtained with RF trained with HC features. Values are shown in terms of %.

Descriptor	A	P	R	S	F1	MCC	BACC
AC	71.76	72.89	84.97	51.47	78.47	39.09	68.22
Haar	62.48	62.65	94.22	13.71	75.26	13.61	53.97
Hist	73.26	77.24	79.19	64.15	78.20	43.66	71.67
HAR	76.78	78.69	84.55	64.84	81.52	50.63	74.69
LBP	79.57	80.74	87.03	68.11	83.77	56.61	77.57
CH_1	78.11	79.99	85.17	67.28	82.50	53.56	76.22
CH_2	78.07	79.84	85.34	66.90	82.50	53.43	76.12
LM	78.25	80.20	85.09	67.73	82.58	53.87	76.41
ZM	65.19	68.16	79.84	42.70	73.54	24.26	61.27

**Table 5:** Performance obtained with SVM trained with deep features. Values are shown in terms of %.

Descriptor	A	P	R	S	F1	MCC	BACC
AlexNet	72.97	77.49	76.56	65.81	77.02	42.80	71.18
DarkNet-19	77.86	82.20	80.82	71.95	81.50	53.28	76.39
DarkNet-53	82.83	85.84	84.99	76.88	85.41	63.65	80.94
DenseNet-201	86.02	89.01	86.61	81.84	87.79	69.91	84.23
EfficientNet B0	82.84	85.76	85.35	76.96	85.55	63.48	81.15
Inception-v3	73.60	78.88	74.73	67.51	76.75	45.86	71.12
Inception-ResNet-v2	69.87	75.03	71.65	62.78	73.29	35.68	67.21
ResNet-18	77.78	82.17	80.82	71.86	81.49	53.13	76.34
ResNet-50	82.77	86.11	84.34	77.87	85.22	63.43	81.11
ResNet-101	82.52	85.76	84.71	76.81	85.23	63.00	80.76
VGG19	79.60	83.94	81.78	73.70	82.84	57.67	77.74
XceptionNet	82.24	85.75	84.22	76.22	84.97	62.53	80.22

**Table 6:** Performance obtained with RF trained with deep features. Values are shown in terms of %.

Descriptor	A	P	R	S	F1	MCC	BACC
AlexNet	84.02	85.55	88.57	77.03	87.03	66.29	82.80
DarkNet-19	88.30	88.68	92.49	81.87	90.54	75.33	87.18
DarkNet-53	90.30	90.72	93.55	85.30	92.11	79.58	89.42
DenseNet-201	91.93	92.61	94.20	88.46	93.40	83.05	91.33
EfficientNet B0	89.89	89.96	93.77	83.92	91.83	78.71	88.85
Inception-v3	85.52	85.64	91.42	76.46	88.44	69.39	83.94
Inception-ResNet-v2	83.25	84.10	89.21	74.10	86.58	64.55	81.65
ResNet-18	86.99	87.32	91.87	79.50	89.53	72.54	85.68
ResNet-50	89.92	90.12	93.63	84.23	91.84	78.77	88.93
ResNet-101	89.59	89.76	93.48	83.62	91.58	78.07	88.55
VGG19	85.98	86.61	90.92	78.40	88.71	70.41	84.66
Xception	88.58	89.08	92.49	82.59	90.75	75.94	87.54

**Table 7:** Performance measures of different classifiers trained with a feature fusion strategy. The classifiers used are DT, SVM, and RF. The strategies compared include combinations of HC and deep features: LBP + DenseNet-201, LBP + EfficientNetB0, DenseNet-201 + EfficientNetB0, and LBP + DenseNet-201 + EfficientNetB0. Values are shown in terms of %.

Strategy	Classifier	A	P	R	S	F1	MCC	BACC
LBP+ DenseNet-201	DT	88.21	89.04	91.84	82.63	90.42	75.16	87.23
	SVM	94.41	95.14	95.66	92.50	95.40	88.29	94.08
	RF	92.16	92.83	94.35	88.80	93.58	83.53	91.57
LBP+ EfficientNetB0	DT	87.55	89.01	90.63	82.82	89.81	73.82	86.72
	SVM	94.05	94.78	95.44	91.92	95.11	87.53	93.68
	RF	89.65	90.19	93.03	84.46	91.59	78.21	88.74
DenseNet-201 + EfficientNetB0	DT	90.30	91.05	93.13	85.94	92.08	79.59	89.54
	SVM	94.89	95.76	95.81	93.49	95.78	89.31	94.65
	RF	91.83	92.31	94.37	87.92	93.33	82.82	91.15
LBP + DenseNet-201 + EfficientNetB0	DT	90.31	91.07	93.13	85.98	92.09	79.63	89.56
	SVM	95.03	95.86	95.93	93.64	95.90	89.59	94.79
	RF	92.26	92.67	94.72	88.50	93.68	83.74	91.61

**Table 8:** Performance measures of the first cross-magnification experiment. Different classifiers were trained on the 160×160 split of the GasHisSDB with a feature fusion strategy and tested on the 120×120 split. The classifiers used were DT, SVM, and RF. Values are shown in terms of %.

Strategy	Classifier	A	P	R	S	F1	MCC	BACC
LBP + DenseNet-201	DT	86.69	90.56	87.68	85.08	89.09	72.10	86.38
	SVM	86.41	97.42	80.20	96.53	87.98	74.51	88.37
	RF	89.04	95.12	86.78	92.74	90.76	77.87	89.76
LBP + EfficientNetB0	DT	85.17	88.66	87.25	81.79	87.95	68.71	84.52
	SVM	85.02	96.81	78.42	95.79	86.65	72.04	87.10
	RF	87.38	90.20	89.36	84.15	89.78	73.30	86.76
DenseNet-201 + EfficientNetB0	DT	88.43	91.65	89.50	86.69	90.56	75.66	88.09
	SVM	85.88	98.40	78.50	97.92	87.33	74.19	88.21
	RF	89.55	94.36	88.43	91.37	91.30	78.51	89.90
LBP + DenseNet-201 + EfficientNetB0	DT	88.42	91.64	89.50	86.67	90.55	75.65	88.08
	SVM	85.82	98.45	78.36	97.98	87.26	74.12	88.17
	RF	89.56	94.79	87.99	92.12	91.26	78.67	90.05

**Table 9:** Performance measures of the second cross-magnification experiment. Different classifiers were trained on the 160×160 split of the GasHisSDB with a feature fusion strategy and tested on the 80×80 split. The classifiers used were DT, SVM, and RF. Values are shown in terms of %.

Strategy	Classifier	A	P	R	S	F1	MCC	BACC
LBP + DenseNet-201	DT	77.05	86.23	73.23	82.70	79.20	54.89	77.97
	SVM	68.92	96.18	49.89	97.07	65.70	49.83	73.48
	RF	78.89	93.29	69.62	92.60	79.73	61.41	81.11
LBP + EfficientNetB0	DT	63.58	89.19	44.34	92.05	59.23	39.09	68.20
	SVM	54.36	96.04	24.53	98.50	39.07	31.44	61.51
	RF	71.38	92.27	56.79	92.96	70.31	50.63	74.88
DenseNet-201 + EfficientNetB0	DT	74.70	88.69	66.02	87.55	75.69	52.89	76.78
	SVM	59.96	96.81	34.02	98.34	50.34	39.00	66.18
	RF	79.73	92.84	71.54	91.84	80.81	62.39	81.69
LBP + DenseNet-201 + EfficientNetB0	DT	74.70	88.69	66.02	87.55	75.69	52.89	76.78
	SVM	60.31	96.72	34.66	98.26	51.03	39.39	66.46
	RF	78.44	93.88	68.33	93.41	79.09	61.10	80.87

**Table 10:** Performance comparison of our work and the previous state-of-the-art works on the GasHisSDB dataset. * indicates that the proposed approach was trained on S-A and directly tested on S-B and S-C without fine-tuning.

Work	Split (%)	Model Details	A (%)		
			S-C	S-B	S-A
[2]	40/40/20	VGG16	96.12	96.47	95.90
	40/40/20	ResNet50	96.09	95.94	96.09
[48]	40/20/40	InceptionV3 trained from scratch	-	-	98.83
	40/20/40	InceptionV3 + ResNet50 (feature concatenation)	-	-	98.80
[14]	60/20/20	LGFFN	-	-	96.81
[49]	80/-/20	MCLNet based on ShuffleNetV2	96.28	97.95	97.85
[6]	40/20/40	Ensemble	97.72	98.68	99.20
Ours	5-fold CVal	SVM with feature fusion	60.31 *	85.82 *	95.03
Ours	5-fold CVal	RF with feature fusion	78.44 *	89.56 *	92.26

## Data Availability

All the material used and developed for this work is available at the following GitHub repository: https://github.com/MurkoZawa/HistopathologyClassification (accessed on 2 July 2024).

## Abbreviations

The following abbreviations are used in this document:

ML	Machine Learning
DL	Deep Learning
HC	Handcrafted
CNN	Convolutional Neural Network
GC	Gastric Cancer
EGC	Early-Stage Gastric Cancer
AGC	Advanced Gastric Cancer
CV	Computer Vision
WSI	Whole Slide Image
LGFFN	Lightweight Gated Fully Fused Network
GHI	Gated Hybrid Input
CH	Chebyshev Moment
LM	Second-Order Legendre Moment
ZM	Zernike Moment
HAR	Rotation-Invariant Haralick
LBP	Local Binary Pattern
Hist	Histogram
AC	Autocorrelogram
Haar	Haar-Like
DT	Decision Tree
kNN	k-Nearest Neighbor
SVM	Support Vector Machine
RF	Random Forest
TN	True Negative
FP	False Positive
FN	False Negative
TP	True Positive
A	Accuracy
P	Precision
R	Recall
S	Specificity
F	F1-Score
MCC	Matthews Correlation Coefficient
BACC	Balanced Accuracy
Cval	Cross-Validation

## Appendix A. Further Results

**Table A1:** Performance obtained with DT trained with HC features. Values are shown in terms of %.

Descriptor	A	P	R	S	F1	MCC	BACC
AC	62.78	69.39	68.97	53.26	69.18	22.20	61.12
Haar	59.70	62.56	83.31	23.43	71.46	8.33	53.37
Hist	68.78	74.49	73.71	61.22	74.10	34.84	67.46
HAR	68.78	74.85	72.99	62.32	73.91	35.10	67.66
LBP	71.22	76.23	76.26	63.47	76.25	39.74	69.87
CH_1	71.11	76.15	76.17	63.35	76.16	39.52	69.76
CH_2	71.05	76.12	76.07	63.35	76.09	39.41	69.71
LM	71.32	76.16	76.64	63.16	76.40	39.87	69.90
ZM	58.06	65.74	64.24	48.57	64.98	12.73	56.40

## Appendix B

**Table A2:** Performance obtained with kNN trained with HC features. Values are shown in terms of %.

Descriptor	A	P	R	S	F1	MCC	BACC
AC	57.34	70.04	51.66	66.06	59.46	17.42	58.86
Haar	42.17	56.99	18.40	78.67	27.82	−3.61	48.53
Hist	64.64	71.97	68.15	59.24	70.01	27.07	63.70
HAR	61.06	68.53	66.05	53.41	67.26	19.29	59.73
LBP	69.51	75.32	73.86	62.82	74.58	36.50	68.34
CH_1	66.16	72.41	71.28	58.29	71.84	29.45	64.78
CH_2	65.46	72.09	70.14	58.29	71.10	28.24	64.21
LM	66.32	72.57	71.38	58.55	71.97	29.81	64.97
ZM	57.40	65.03	64.19	46.97	64.60	11.12	55.58

**Table A3:** Performance obtained with DT trained with deep features. Values are shown in terms of %.

Descriptor	A	P	R	S	F1	MCC	BACC
AlexNet	75.00	79.47	79.19	68.57	79.33	47.72	73.88
DarkNet-19	78.25	82.04	82.04	72.42	82.04	54.46	77.23
DarkNet-53	81.64	84.78	84.95	76.57	84.86	61.55	80.76
DenseNet-201	84.92	87.51	87.60	80.80	87.56	68.42	84.20
EfficientNet B0	78.79	82.62	82.29	73.41	82.46	55.64	77.85
Inception-v3	74.54	79.21	78.60	68.30	78.90	46.81	73.45
Inception-ResNet-v2	73.52	78.02	78.35	66.10	78.18	44.49	72.22
ResNet-18	76.73	81.20	80.13	71.50	80.66	51.46	75.82
ResNet-50	81.30	84.62	84.47	76.42	84.55	60.87	80.45
ResNet-101	80.13	83.67	83.48	74.97	83.58	58.42	79.23
VGG19	76.40	80.97	79.79	71.20	80.37	50.80	75.49
Xception	79.38	83.30	82.49	74.59	82.89	56.94	78.54

**Table A4:** Performance obtained with kNN trained with deep features. Values are shown in terms of %.

Descriptor	A	P	R	S	F1	MCC	BACC
AlexNet	80.85	83.80	84.77	74.82	84.28	59.78	79.80
DarkNet-19	83.99	86.47	87.20	79.05	86.84	66.40	83.13
DarkNet-53	88.25	88.89	92.11	82.32	90.48	75.25	87.22
DenseNet-201	88.21	89.04	91.84	82.63	90.42	75.16	87.23
EfficientNet B0	87.53	89.01	90.60	82.82	89.80	73.79	86.71
Inception-ResNet-v2	76.13	79.89	80.98	68.69	80.43	49.85	74.83
Inception-v3	80.88	83.65	85.04	74.48	84.34	59.80	79.76
ResNet-101	87.89	88.57	91.87	81.79	90.19	74.48	86.83
ResNet-18	84.21	85.89	88.47	77.68	87.16	66.73	83.07
ResNet-50	86.97	88.20	91.09	80.41	89.62	73.19	85.75
VGG19	83.35	85.94	86.94	77.95	86.44	65.88	82.45
Xception	85.15	86.74	89.44	78.95	88.07	70.17	84.20

## References

[1] Ilic M., Ilic I. (2022). Epidemiology of stomach cancer. World J. Gastroenterol..

[2] Hu W., Li C., Li X., Rahaman M.M., Ma J., Zhang Y., Chen H., Liu W., Sun C., Yao Y. (2022). GasHisSDB: A new gastric histopathology image dataset for computer aided diagnosis of gastric cancer. Comput. Biol. Med..

[3] Hirasawa T., Aoyama K., Tanimoto T., Ishihara S., Shichijo S., Ozawa T., Ohnishi T., Fujishiro M., Matsuo K., Fujisaki J. (2018). Application of artificial intelligence using a convolutional neural network for detecting gastric cancer in endoscopic images. Gastric Cancer.

[4] Zhao Y., Hu B., Wang Y., Yin X., Jiang Y., Zhu X. (2022). Identification of gastric cancer with convolutional neural networks: A systematic review. Multim. Tools Appl..

[5] Xie K., Peng J. (2023). Deep learning-based gastric cancer diagnosis and clinical management. J. Radiat. Res. Appl. Sci..

[6] Yong M.P., Hum Y.C., Lai K.W., Lee Y.L., Goh C.H., Yap W.S., Tee Y.K. (2023). Histopathological gastric cancer detection on GasHisSDB dataset using deep ensemble learning. Diagnostics.

[7] Yoon H.J., Kim S., Kim J.H., Keum J.S., Oh S.I., Jo J., Chun J., Youn Y.H., Park H., Kwon I.G. (2019). A lesion-based convolutional neural network improves endoscopic detection and depth prediction of early gastric cancer. J. Clin. Med..

[8] Hu W., Chen H., Liu W., Li X., Sun H., Huang X., Grzegorzek M., Li C. (2022). A comparative study of gastric histopathology sub-size image classification: From linear regression to visual transformer. Front. Med..

[9] Zhang K., Wang H., Cheng Y., Liu H., Gong Q., Zeng Q., Zhang T., Wei G., Wei Z., Chen D. (2024). Early gastric cancer detection and lesion segmentation based on deep learning and gastroscopic images. Sci. Rep..

[10] Marini N., Otálora S., Podareanu D., van Rijthoven M., van der Laak J., Ciompi F., Müller H., Atzori M. (2021). Multi_Scale_Tools: A Python Library to Exploit Multi-Scale Whole Slide Images. Front. Comput. Sci..

[11] Ashtaiwi A. (2022). Optimal Histopathological Magnification Factors for Deep Learning-Based Breast Cancer Prediction. Appl. Syst. Innov..

[12] Cao R., Tang L., Fang M., Zhong L., Wang S., Gong L., Li J., Dong D., Tian J. (2022). Artificial intelligence in gastric cancer: Applications and challenges. Gastroenterol. Rep..

[13] Hu W., Li C., Rahaman M.M., Chen H., Liu W., Yao Y., Sun H., Grzegorzek M., Li X. (2023). EBHI: A new Enteroscope Biopsy Histopathological H&E Image Dataset for image classification evaluation. Phys. Medica.

[14] Li S., Liu W. (2022). LGFFN-GHI: A Local-Global Feature Fuse Network for Gastric Histopathological Image Classification. J. Comput. Commun..

[15] Putzu L., Loddo A., Ruberto C.D., Tsapatsoulis N., Panayides A., Theocharides T., Lanitis A., Pattichis C.S., Vento M. (2021). Invariant Moments, Textural and Deep Features for Diagnostic MR and CT Image Retrieval. Proceedings of the 19th International Conference of Computer Analysis of Images and Patterns, CAIP 2021.

[16] Ruberto C.D., Loddo A., Putzu L. (2023). On The Potential of Image Moments for Medical Diagnosis. J. Imaging.

[17] Mukundan R., Ong S.H., Lee P.A. (2001). Image analysis by Tchebichef moments. IEEE Trans. Image Process..

[18] Ruberto C.D., Putzu L., Rodriguez G. (2018). Fast and accurate computation of orthogonal moments for texture analysis. Pattern Recognit..

[19] Teh C., Chin R.T. (1988). On Image Analysis by the Methods of Moments. IEEE Trans. Pattern Anal. Mach. Intell..

[20] Teague M.R. (1980). Image analysis via the general theory of moments. J. Opt. Soc. Am..

[21] Wee C., Raveendran P. (2007). On the computational aspects of Zernike moments. Image Vis. Comput..

[22] Mirjalili F., Hardeberg J.Y. (2022). On the Quantification of Visual Texture Complexity. J. Imaging.

[23] Putzu L., Ruberto C.D., Battiato S., Gallo G., Schettini R., Stanco F. (2017). Rotation Invariant Co-occurrence Matrix Features. Proceedings of the 19th International Conference of Image Analysis and Processing, ICIAP 2017.

[24] He D.C., Wang L. (1990). Texture unit, texture spectrum, and texture analysis. IEEE Trans. Geosci. Remote. Sens..

[25] Ojala T., Pietikäinen M., Mäenpää T. (2002). Multiresolution Gray-Scale and Rotation Invariant Texture Classification with Local Binary Patterns. IEEE Trans. Pattern Anal. Mach. Intell..

[26] Van de Weijer J., Schmid C., Leonardis A., Bischof H., Pinz A. (2006). Coloring Local Feature Extraction. Proceedings of the 9th European Conference on Computer Vision, ECCV 2006.

[27] Huang J., Kumar R., Mitra M., Zhu W., Zabih R. (1997). Image Indexing Using Color Correlograms. Proceedings of the 1997 IEEE Conference on Computer Vision and Pattern Recognition (CVPR ’97).

[28] Viola P.A., Jones M.J. (2001). Rapid Object Detection using a Boosted Cascade of Simple Features. Proceedings of the 2001 IEEE Computer Society Conference on Computer Vision and Pattern Recognition, (CVPR 2001).

[29] Bodapati J.D., Veeranjaneyulu N. (2019). Feature Extraction and Classification UsingDeep Convolutional Neural Networks. J. Cyber Secur. Mobil..

[30] Deng J., Dong W., Socher R., Li L., Li K., Fei-Fei L. (2009). ImageNet: A large-scale hierarchical image database. Proceedings of the 2009 IEEE Computer Society Conference on Computer Vision and Pattern Recognition, (CVPR 2009).

[31] Putzu L., Piras L., Giacinto G. (2020). Convolutional neural networks for relevance feedback in content based image retrieval. Multim. Tools Appl..

[32] Wang H., Wu X., Huang Z., Xing E.P. (2020). High-Frequency Component Helps Explain the Generalization of Convolutional Neural Networks. Proceedings of the 2020 IEEE/CVF Conference on Computer Vision and Pattern Recognition, CVPR 2020.

[33] Krizhevsky A., Sutskever I., Hinton G.E. (2017). ImageNet classification with deep convolutional neural networks. Commun. ACM.

[34] Redmon J., Farhadi A. (2017). YOLO9000: Better, Faster, Stronger. Proceedings of the 2017 IEEE Conference on Computer Vision and Pattern Recognition, CVPR 2017.

[35] Redmon J., Farhadi A. (2018). YOLOv3: An Incremental Improvement. arXiv.

[36] Huang G., Liu Z., van der Maaten L., Weinberger K.Q. (2017). Densely Connected Convolutional Networks. Proceedings of the 2017 IEEE Conference on Computer Vision and Pattern Recognition, CVPR 2017.

[37] Tan M., Le Q.V., Chaudhuri K., Salakhutdinov R. (2019). EfficientNet: Rethinking Model Scaling for Convolutional Neural Networks. Proceedings of the 36th International Conference on Machine Learning, ICML 2019.

[38] Szegedy C., Vanhoucke V., Ioffe S., Shlens J., Wojna Z. (2016). Rethinking the Inception Architecture for Computer Vision. Proceedings of the 2016 IEEE Conference on Computer Vision and Pattern Recognition, CVPR 2016.

[39] Szegedy C., Ioffe S., Vanhoucke V., Alemi A.A., Singh S., Markovitch S. (2017). Inception-v4, Inception-ResNet and the Impact of Residual Connections on Learning. Proceedings of the 31st AAAI Conference on Artificial Intelligence.

[40] He K., Zhang X., Ren S., Sun J. (2016). Deep Residual Learning for Image Recognition. Proceedings of the 2016 IEEE Conference on Computer Vision and Pattern Recognition, CVPR 2016.

[41] Simonyan K., Zisserman A., Bengio Y., LeCun Y. (2015). Very Deep Convolutional Networks for Large-Scale Image Recognition. Proceedings of the 3rd International Conference on Learning Representations, ICLR 2015.

[42] Chollet F. (2017). Xception: Deep Learning with Depthwise Separable Convolutions. Proceedings of the 2017 IEEE Conference on Computer Vision and Pattern Recognition, CVPR 2017.

[43] Ioffe S., Szegedy C., Bach F.R., Blei D.M. (2015). Batch Normalization: Accelerating Deep Network Training by Reducing Internal Covariate Shift. Proceedings of the 32nd International Conference on Machine Learning, ICML 2015.

[44] Quinlan J.R. (1983). Learning efficient classification procedures and their application to chess end games. Machine Learning.

[45] Cover T.M., Hart P.E. (1967). Nearest neighbor pattern classification. IEEE Trans. Inf. Theory.

[46] Lin Y., Lv F., Zhu S., Yang M., Cour T., Yu K., Cao L., Huang T.S. (2011). Large-scale image classification: Fast feature extraction and SVM training. Proceedings of the The 24th IEEE Conference on Computer Vision and Pattern Recognition, CVPR 2011.

[47] Breiman L. (2001). Random Forests. Mach. Learn..

[48] Springenberg M., Frommholz A., Wenzel M., Weicken E., Ma J., Strodthoff N. (2023). From modern CNNs to vision transformers: Assessing the performance, robustness, and classification strategies of deep learning models in histopathology. Med. Image Anal..

[49] Fu X., Liu S., Li C., Sun J. (2023). MCLNet: An multidimensional convolutional lightweight network for gastric histopathology image classification. Biomed. Signal Process. Control..

[50] Song Y., Wang T., Cai P., Mondal S.K., Sahoo J.P. (2023). A comprehensive survey of few-shot learning: Evolution, applications, challenges, and opportunities. ACM Comput. Surv..

